# Respiratory Muscle Strengths and Their Association with Lean Mass and Handgrip Strengths in Older Institutionalized Individuals

**DOI:** 10.3390/jcm9092727

**Published:** 2020-08-24

**Authors:** Francisco Miguel Martínez-Arnau, Cristina Buigues, Rosa Fonfría-Vivas, Omar Cauli

**Affiliations:** 1 Department of Physiotherapy, University of Valencia, 46010 Valencia, Spain; Francisco.m.martinez@uv.es; 2 Frailty and Cognitive Impairment Research Group (FROG), University of Valencia, 46010 Valencia, Spain; cristina.buigues@uv.es (C.B.); rosa.fonfria@uv.es (R.F.-V.); 3 Department of Nursing, University of Valencia, 46010 Valencia, Spain

**Keywords:** spirometry, urea, fatigue, respiratory system, skeletal muscles, lipids, transaminases

## Abstract

The study of reduced respiratory muscle strengths in relation to the loss of muscular function associated with ageing is of great interest in the study of sarcopenia in older institutionalized individuals. The present study assesses the association between respiratory muscle parameters and skeletal mass content and strength, and analyzes associations with blood cell counts and biochemical parameters related to protein, lipid, glucose and ion profiles. A multicenter cross-sectional study was performed among patients institutionalized in nursing homes. The respiratory muscle function was evaluated by peak expiratory flow, maximal respiratory pressures and spirometry parameters, and skeletal mass function and lean mass content with handgrip strength, walking speed and bioimpedance, respectively. The prevalence of reduced respiratory muscle strength in the sample ranged from 37.9% to 80.7%. Peak expiratory flow significantly (*p* < 0.05) correlated to handgrip strength and gait speed, as well as maximal inspiratory pressure (*p* < 0.01). Maximal expiratory pressure significantly (*p* < 0.01) correlated to handgrip strength. No correlation was obtained with muscle mass in any of parameters related to reduced respiratory muscle strength. The most significant associations within the blood biochemical parameters were observed for some protein and lipid biomarkers e.g., glutamate-oxaloacetate transaminase (GOT), urea, triglycerides and cholesterol. Respiratory function muscle parameters, peak expiratory flow and maximal respiratory pressures were correlated with reduced strength and functional impairment but not with lean mass content. We identified for the first time a relationship between peak expiratory flow (PEF) values and GOT and urea concentrations in blood which deserves future investigations in order to manage these parameters as a possible biomarkers of reduced respiratory muscle strength.

## 1. Introduction

Sarcopenia is a geriatric syndrome that according to the European Working Group on Sarcopenia in Older People (EWGSOP) guidelines, is defined as a progressive and generalized loss of skeletal muscle mass and strength, with a risk of adverse outcomes, such as functional capacity impairment, dependence, falls and fractures, negative impact on quality of life, hospitalization and death [1]. In older individuals, sarcopenia has a widespread effect on all skeletal muscles throughout the body, but the features of sarcopenia in the respiratory muscles and its relationship with established sarcopenia parameters such as reduced lean mass, poor muscular strength and functional impairment [1,2] have been less widely investigated in older individuals [3,4], and no studies have been performed in nursing home residents, a significant population in western societies with a huge burden of comorbidities, including sarcopenia [5,6,7,8]. Besides the loss of muscular mass and strength, aging leads to proteolysis of elastic fiber and an increase in collagen in the pulmonary parenchyma, which coupled with an increase in the rigidity of the chest wall generates a mechanical disadvantage, and weakness of the respiratory muscles over time [9,10]. These changes results in a diminished respiratory muscle strength (RMS), referred to as sarcopenia of the respiratory muscle or reduced respiratory muscle strength as just it is analysed by quantifying the decline in respiratory function [3]. Respiratory muscles are also responsible of producing a proper pressure difference between inspiration and expiration to generate a correct airway flow rate, which guarantees a good respiratory function [11]. Other respiratory parameters, such as vital capacity (VC), forced vital capacity (FVC), forced expiratory volume in 1 s (FEV1), and peak expiratory flow rate (PEF) are also affected as a result of changes in elastic recoil and thorax compliance associated with aging [3,11,12]. RMS is therefore related to FEV1, FVC, and PEF. Even in patients without airway obstruction, these functions may decline due to age-induced weakness of the respiratory muscles. PEF measurements were recommended over RMS measurements for the assessment of respiratory function in the EWGSOP consensus report published in 2010 [2]. However, the EWGSOP report also indicated that PEF measurements should be used in association with other assessments, because there is a limited evidence about the relationship between PEF and skeletal muscle mass/sarcopenia in older adults. A previous study revealed that PEF is a significant predictor of mortality in older adults [13,14]. Further studies have demonstrated that sarcopenia is related to an increased incidence of pulmonary complications after surgery [15,16,17] and aspiration pneumonia mortality [18]. Izawa et al. [19] evaluated the relationship between maximal inspiratory pressure (MIP) and physical function as a measure of sarcopenia in older patients with heart disease, and found that sarcopenic patients presented lower values of MIP which also correlated with reduced skeletal muscle mass index, gait speed and hand grip strength. There is a lack of studies demonstrating the association between respiratory muscle weakness and sarcopenia parameters (reduced lean mass and muscular strength and low physical performance) in older institutionalized individuals. Moreover, no studies about the relationship of respiratory muscle function and blood analytical parameters in sarcopenic individuals have been performed. The objectives in this study were therefore to compare respiratory muscle function with lean mass content, handgrip strength and functional impairment (walking speed) in order to assess whether there is an association between respiratory muscle parameters such as the maximum respiratory pressures and peak expiratory flow and parameters of skeletal muscular function. Since skeletal sarcopenia have been associated to malnutrition and undernutrition, which in turn is accompanied by several alterations detectable in blood regarding both blood cell counts and biochemical metabolic markers [20,21,22,23] we also evaluated the associations between the parameters related to respiratory muscle strength and skeletal sarcopenia with blood cell counts and biochemical parameters related to protein, lipid, glucose and indirectly with energy production (glucose, creatinine, transaminases, and ions concentrations).

## 2. Materials and Methods

### 2.1. Design and Study Population

A cross-sectional study was conducted in individuals institutionalized in nursing homes and long-stay centers for the older individuals in the province of Valencia, Spain (GeroResidencias La Saleta, Valencia). We selected nursing home residents of both genders. Participants were excluded if they were unable to understand the content of questionnaires (moderate-severe cognitive impairment), had a poorly controlled major psychiatric disease (schizophrenia, bipolar disorders, etc.), acute infections, or a known cancer condition. According to the requirements of the Declaration of Helsinki, written consent was obtained from all of the selected subjects before beginning the study, after informing them about the procedures involved and the purpose of the research. The entire study protocol was approved by the local ethical committee at the University of Valencia (H1524420647893, approved 5 July 2018).

### 2.2. Sociodemographic and Clinical Variables

Socio-demographic variables and medical conditions were recorded, including the number of medications taken, the type and number of any comorbidities using the Charlson index, and several hematological and biochemical parameters. The Charlson index was used to assess comorbidity (with a Cronbach’s Alpha of 0.78) [24]. This index assesses 16 diseases that are explicitly defined and scored by a continuous variable from 0 to 31. With this index, the 10-year survival prediction is estimated for patients with comorbidity [25].

### 2.3. Measurement of Respiratory Muscle Function

The assessment of respiratory function was carried out through two different tests, the assessment of lung volumes and flows by performing a forced spirometry, and the assessment of the maximum respiratory pressures that the respiratory muscles are capable of generating at mouth level as a result of maximum effort.

The spirometric assessment followed the standardized recommendations of the European Respiratory Society [26]. The patient was placed in a seated position, with his back supported by the backrest and with nasal clamps to avoid air leakage. The maneuver was explained in detail to the patient to minimize errors, requesting an initial maximum inspiration to reach total lung capacity, which allows the subsequent performance of a forced maximum expiration for at least 6 s, until the limit of expiration is reached. At least three manoeuvres are performed, with a rest of 1 min between each one, and the highest value of the three repetitions is recorded.

By carrying out this test, the following volumes and forced pulmonary capacities in absolute and relative values were obtained: forced vital capacity (FVC), forced expiratory volume in the first second (FEV1), FEV1/FVC, forced expiratory volume in smaller than 1mm diameter tracks (FEV2575) and peak expiratory flow (PEF). At least three repetitions of the maneuver were performed (with a maximum of 8 repetitions) to achieve the correct execution of the test, discarding those spirometric maneuvers with artifacts in their performance or variations of more than 0.150 L between the highest FEV1 and/or FVC values, as recommended by the ATS/ERS [26].

For the assessment of respiratory muscle strength, maximum static respiratory pressures in the mouth, inspiratory (MIP) and expiratory (MEP) were measured. These parameters allow us to know in a simple way the global force that the respiratory muscles are capable of exerting. The tests require the collaboration of the patient to perform a maximum isometric effort. The standardized regulations for this test were followed [27,28]. To evaluate the MIP, the patient was instructed to start from the residual volume and for the MEP to start from the total lung capacity, so that the maximum value of the three maneuvers could be collected, with a variation of less than 10% between them and a 1-min pause between each of the repetitions. This excluded those attempts where there was more than 10% variation between them, as recommended by Laveneziana, et al. [28].The proposed cut-off points for PEF and maximum respiratory pressures (MIP and MEP) were used to establish the existence of respiratory sarcopenia. The cut-off point for PEF was set at 4.40 L/s for men and 3.21 L/s for women [22]. The cut-off point for MIP was set at less than or equal to 55 H_2_O cm for men and less than or equal to 45 H_2_O cm for women, while for MEP it was set at less than or equal to 60 H_2_O cm for men and less than or equal to 50 H_2_O cm for women [4]. Before the test was conducted, the steps for correctly performing the test were carefully explained to the participants. Once explained, a test of all the steps to be followed was carried out, without demanding maximum effort from the participants to avoid accumulated fatigue. Afterwards, the tests were carried out in accordance with international standards [28].

The older institutionalized population has a high prevalence of cognitive impairment, which could make this type of testing difficult. However, we excluded patients with moderate and severe cognitive impairment, so that the collaboration of patients included was adequate to perform these tests. In addition, an adaptation procedure was carried out on the study subjects before the definitive test, excluding from the sample those subjects who presented poor coordination and, therefore, difficulty in carrying out the test at the discretion of the evaluator. In all the centres, assessments were made in the morning between 8 and 11 am and in the same period of time. To avoid inter-observer errors, all measurements were taken by the same trained investigator.

In addition, to analyze reliability, we assessed the stability of the measure obtaining values of intraclass correlation coefficient (ICC; one-way, mixed-effects model) between PEF values in the three centers of 0.71, what was indicative of moderate to good reliability.

### 2.4. Measurement of Sarcopenia

Muscle skeletal sarcopenia was assessed by indirect measures of muscle function and muscle mass, such as handgrip strength assessed by hand-dynamometry, walking speed and bioimpedance respectively. Hand-dynamometer was assessed in the dominant hand by means of a JAMAR dynamometer (Lafayette Instrument Company, Lafayette, IN, USA) as previously described [29]. The subject was placed in a standard position: in a sitting position, with the shoulder at 0° of flexion, the elbow attached to the body at 90° of flexion and the forearm in a neutral position. After the subject is positioned appropriately, the examiner asks the patient to squeeze as hard as possible for 3 s and then relax. Three attempts were made, with 1 min rest in between. The mean value obtained was recorded. The cut-offs for handgrip strength were ≤30 kg/m^2^ for men and ≤20 kg/m^2^ for women [2]. The walking speed was assessed using the 4-m walking test [30]. The patient was asked to walk at usual pace and from a standing start and using their usual walking aid. The time required to cover this distance was recorded and, based on this, the walking speed in m/s was calculated. The cut-off for low walking speed was ≤0.8 m/s walking through 4 m [2]. The body composition was assessed by bioelectrical impedance analysis (BIA) with a BF-300 device (Tanita, Tokyo, Japan) as previously described [31,32]. The BIA measure was performed with a standard technique using a single frequency of 50 KHz and 550 mA, and the placement of four electrodes in a distal position (four electrodes at feet) while participant was in a standing position. BIA measurements were carried out in the early morning following the next considerations: (1) No physical exercise in the previous hours; (2) 2–3 h of fasting, including drinking plenty of water or alcohol; (3) urination 30 min before the test; (4) no metal parts at the time of the test. The values of reactance and resistance were then recorded once the patient was stabilized. The repeatability and accuracy of the resistance and reactance measurements enabled the smallest changes to be recorded to a resolution of 0.1 Ω. Muscle mass was calculated using the formula of Janssen et al. [31]: muscle mass (kg) = [(height^2^/R × 0.401) + (3.825 × sex) + (−0.701 × age) + 5.102] where height is expressed in cm, R in Ω, age in years and female sex has a value of zero and males a value of one. The muscle mass index (MMI) is defined as the muscle mass a person has, corrected by body surface area (muscle mass/height^2^). The bioimpedance test was performed early in the morning while the patient is at rest, after overnight fasting (food and drink) and removing all metal elements. The cut-off for the loss of muscle mass assessed by bioimpedance of the whole body were ≤5.5 kg/m^2^ for women and ≤7.25 kg/m^2^) for men [2]. These muscle mass values are adjusted with the cut-off values for the Spanish population being 8.31 kg/m^2^ for men and 6.68 kg/m^2^ for women [33]. In order to minimize the influence of physical performance across the time of the day, all measurements were always conducted between 8–11 a.m.

### 2.5. Haemogram and Analytical Parameters

To obtain the analytical determinations, the usual blood controls carried out in residential centers were used. Thus, blood samples were collected from each subject at approximately 8 am (after 8–10 h fasting). 10 mL of blood plasma was collected into Vacutainer tubes (BD, Franklin Lakes, NJ, USA) containing EDTA.

Clinical laboratories belonging to local public health centers were used to analyze the different hematological parameters (white blood cells, hemoglobin, erythrocytes, and platelets) and biochemical parameters (glucose, urea, urate, cholesterol, triglycerides, creatinine, glutamic oxaloacetic transaminase [GOT], and serum glutamic pyruvic transaminase [GPT], sodium ions [Na^+^], potassium ions [K^+^], and Calcium [Ca^++^]). Within public health centers, the variation range of metabolites in plasma sample varies between 0.4–1.1% dependent on the metabolite.

### 2.6. Statistical Analysis

Quantitative variables were analysed using descriptive statistics, specifically central tendency measures (means), standard error of the mean (SEM), 95% confidence interval and ranges. Frequencies and percentages were used to describe the qualitative variables. The normal distribution of the variables, in order to determine whether to carry out parametric or non-parametric tests, was analysed using the Shapiro-Wilk test. Outliers were identified on the boxplot drawn in SPSS program which uses a step of 1.5 × IQR (Interquartile range). No outliers were identified and all data were included in the statistical analysis. Differences in quantitative variables between the two groups were analyzed with the two-tailed tests e.g., parametric Student t-test or the nonparametric Mann-Whitney U-test. To analyze the correlation between quantitative variables, the parametric Pearson test or the non-parametric Spearman’s test was used depending on their distribution. Statistical significance was considered at *p* < 0.05. SPSS version 25.0 statistical package (SPSS Inc., Chicago, IL, USA) was used to perform the statistical analyses.

## 3. Results

### 3.1. Sociodemographic and Clinical Parameters of the Study Sample

A total of 58 subjects (67.2% female) living in three nursing care centers located in the province of Valencia (Spain) were enrolled in the study (Table 1). All the participants were Caucasian. Their age ranged from 55 to 93 years, and the mean age was 78.6 ± 8.9 years. 63.8% of the subjects were independent in their walking ability (they did not require external aids such as a cane or walker). Smokers were 15.5% (*n* = 9) of the sample. A percentage of 21.1% (*n* = 12) in the study sample used bronchodilators as a usual treatment. Among individuals using bronchodilators, *n* = 6 used bronchodilator therapy containing glucocorticoids. Regarding the use of common medications affecting the muscular system, none of the individuals received oral glucocorticoid treatment, 37.9% (*n* = 22) used statins to lower cholesterol levels and 5.2% (*n* = 3) used muscle relaxant drugs. Mean body mass index was 28.8 ± 5.8 (Range 18.7–50.2). The Charlson comorbidity index score adjusted for age was 5.4 ± 1.9 (Range 1.0–11.0). The occurrence of the most common comorbidities are indicated in Table 1.

Respiratory function assessment showed an absence of respiratory failure related to oxyhemoglobin saturation, with 95.9 ± 1.9% (range 91.0–99.0). Respiratory functional exploration showed spirometric values within normal ranges for a population of these characteristics (FVC at 84.0 ± 23.6% (Range 23.0–149.0) and FEV1 at 83.3 ± 28.3% (Range 20.0–160.0)), except for a small reduction in the permeability of the smaller diameter airway, with an FEV2575 at 54.5 ± 25.7% (Range 12.0–149.0). Respiratory muscle strength was diminished, at both inspiratory (36.5 ± 17.4 H_2_O cm) and expiratory (58.9 ± 23.7 H_2_O cm) levels. The maximal respiratory pressures (MIP and MEP) and spirometric parameter values (FVC, FEV1, FEV1/FVC, FEV2575 and PEF) are shown in Table 2.

A positive correlation was found between oxyhemoglobin saturation and FVC (r = 0.287 *p* = 0.034, Pearson test) and oxyhemoglobin saturation and FEV1 (r = 0.269 *p* = 0.047, Pearson test). No correlations were found between heart rate and any other respiratory parameters.

A positive correlation can be found between the various parameters that describe the spirometric function by analyzing the correlation between the different parameters of respiratory function. There was a significant correlation between FVC percentage values and FEV1 percentage values (r = 0.894, *p* < 0.001, Pearson test), FEV2575 percentage values (r = 0.473, *p* < 0.001, Pearson test) and PEF (r = 0.281 *p* = 0.033, Pearson test). Significant correlations were also found between FEV1 percentage values and FEV2575 percentage values (r = 0.689, *p* < 0.001, Pearson test). There was a correlation between PEF and maximum respiratory pressures, with both MIP (r = 0.419, *p* < 0.001, Pearson test) and with MEP (r = 0.575, *p* < 0.001, Pearson test), and the maximum respiratory pressures between them (r = 0.559, *p* < 0.001, Pearson test).

Based on the PEF cut-off points established by Kera et al., (22), the prevalence of respiratory sarcopenia in the sample studied was 70.7%. On the other hand, if the values of MIP and MEP established by Ohara et al., (4) are taken as the benchmark, the prevalence of respiratory sarcopenia was 80.7% and 37.9%, respectively.

### 3.2. Evaluation of Skeletal Muscle Mass and Function

According to the EWGSOP guidelines, 17.6% of the subjects were classified as sarcopenic, with 17.6% meeting the criteria of reduced lean mass, 65.4% meeting the criteria of low physical performance and 84.5% meeting the criteria of reduced muscle strength. The mean values of each criterion were skeletal muscle-mass index of 9.21 ± 2.793 kg/m^2^, walking speed of 0.66 ± 0.331 m/s and handgrip strength of 17.90 ± 8.506 kg. The data from the anthropometric characteristics of all the participants in this study are summarized in Table 3.

### 3.3. Evaluation of the Relationship between Muscle Skeleñata Parameters (Mass and Function) and Muscle Respiratory Function 

There was a significant and positive correlation between physical performance and PEF absolute values (r = 0.563, *p* < 0.001, Spearman’s test), PEF percentage values (r = 0.440, *p* = 0.001, Pearson test) and MIP values (r = 0.354, *p* = 0.011, Spearman’s test). No correlation between physical performance and MEP was found (r = 0.268, *p* = 0.268, Spearman’s test). No significant correlation was found between the other parameters of respiratory function and physical performance (*p* > 0.05 in all cases).

**Figure 1:**
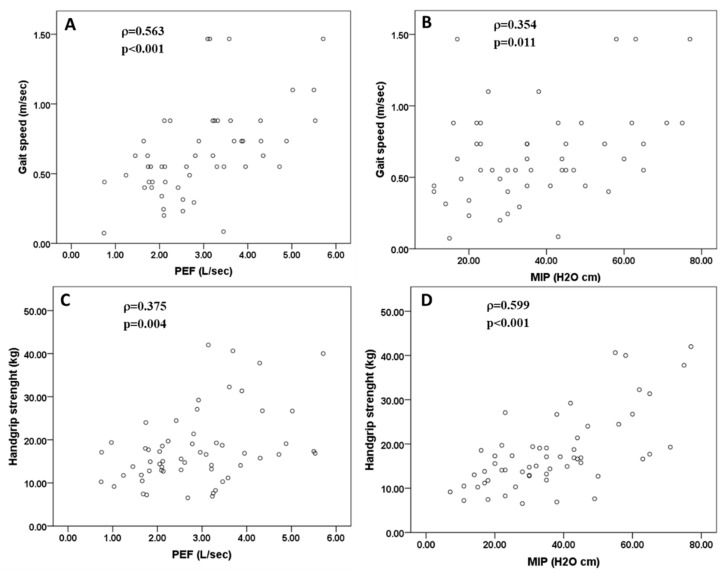
Representation of the significant correlations between skeletal and respiratory muscle sarcopenia parameters. Significant correlations between gait speed and PEF (**A**) or MIP (**B**) and between handgrip strength and PEF (**C**) or MIP (**D**).

There was a significant and positive correlation between handgrip strength and MIP values (r = 0.599, *p* < 0.001, Spearman’s test), MEP values (r = 0.465, *p* < 0.001, Spearman’s test) and PEF absolute values (r = 0.375, *p* = 0.004, Spearman’s test). There was also a significant but negative correlation between handgrip strength and FEV1 percentage values (r = −0.307, *p* = 0.019, Spearman’s test). No significant correlation was found between other parameters of respiratory function and handgrip strength (*p* > 0.05 in all cases) (Figure 1).

No significant correlations were found between skeletal muscle mass index and respiratory function parameters, in relation to either PEF absolute values (r = 0.252, *p* = 0.074, Spearman’s test), or MIP (r = 0.143, *p* = 0.322, Spearman’s test), or MEP (r = 0.225, *p* = 0.112, Spearman’s test).

**Figure 2:**
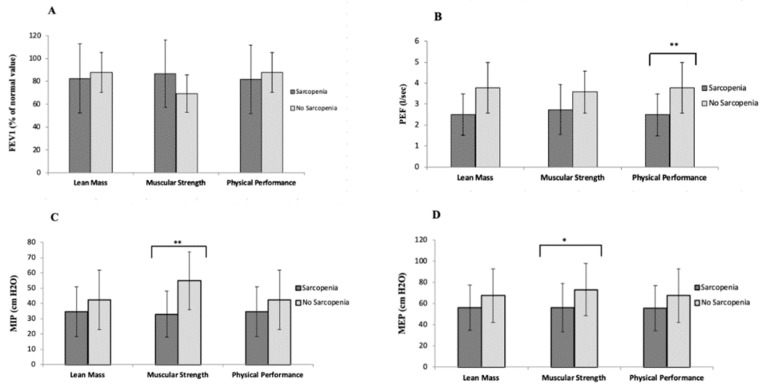
Mean difference of respiratory parameters ((**A**): FEV1; (**B**): PEF; (**C**): MIP; (**D**): MEP) according to the presence or not of the three cut-off values for sarcopenia parameters * *p* < 0.05; ** *p* < 0.001.

We categorized patients based on cut-off scores for skeletal sarcopenia (see Methods section) and we evaluated whether there were any differences in the respiratory parameters and respiratory muscle parameters (Figure 2).

As for physical performance, differences were observed in both PEF (NS = 3.78 vs. S = 2.49, MeanDiff = 1.29 [95%CI: 0.67–1.91], *p* < 0.001) and PEF% (NS = 64.11 vs. S = 47.21, MeanDiff = 16.90 [95%CI: 6.59–27.22], *p* = 0.002).

For the handgrip strength, different maximal respiratory pressures were observed in both groups, MIP (NS = 54.89 vs. S = 33.06, MeanDiff = 21.83 [95%CI: 10.48–33.18], *p* < 0.001) and MEP (NS = 73.22 vs. S = 56.69, MeanDiff = 16.92 [95%CI: 0.13–37.70], *p* = 0.048). When analyzing the PEF we observed no statistically significant differences, although a trend was observed in them (NS = 3.57 vs. S = 2.74, MeanDiff = 0.86 [95%CI: −0.006–1.72], *p* = 0.052) 

No significant differences for lean mass content were observed for any of the comparisons (*p* > 0.05) (Figure 2).

**Figure 3:**
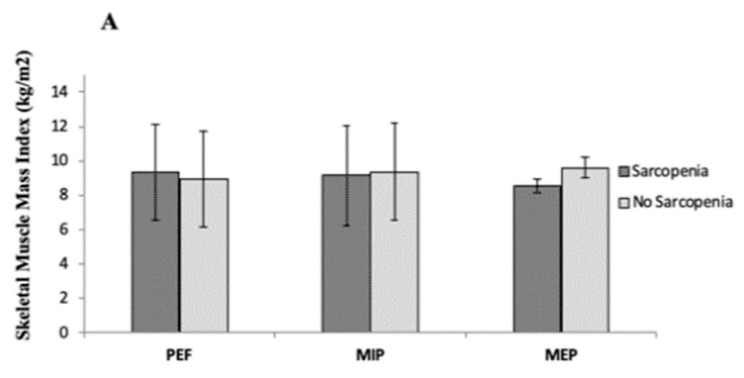

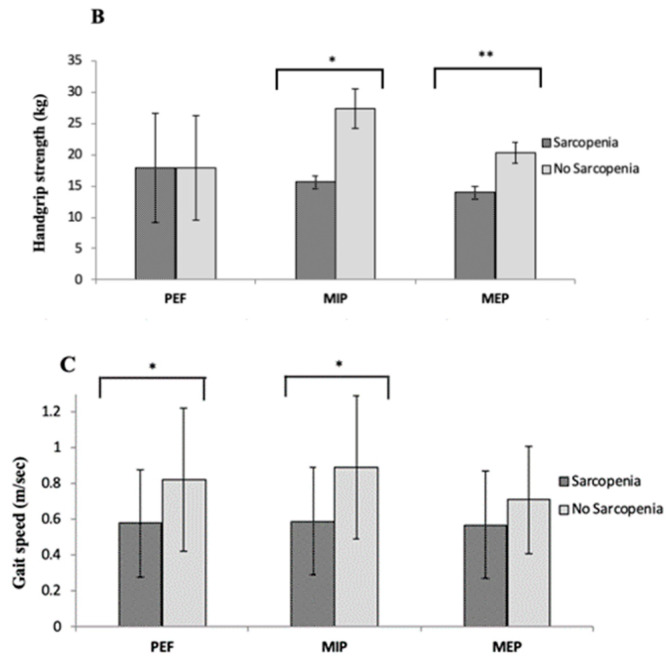
Mean difference of muscle mass (**A**), Handgrip strength (**B**) and gait speed (**C**) according to the presence of each respiratory muscle sarcopenia criteria. * *p* < 0.05; ** *p* < 0.001.

We also categorized patients based on respiratory muscle sarcopenia according to Kera et al. (22) and Ohara et al. (4) (see methods), and we evaluated whether there were any differences in the somatic sarcopenia parameters, such as skeletal muscle mass index, handgrip strength and gait speed (Figure 3).

For MIP, differences were observed in both gait speed (NS = 0.89 vs. S = 0.59, MeanDiff = 0.30 [95%CI: 0.51–0.85], *p* = 0.007) and handgrip strength (NS = 27.35 vs. S = 15.64, MeanDiff = 11.71 [95%CI: 4.75–18.66], *p* = 0.003). No differences were found for skeletal muscle mass index (*p* = 0.844).

As regards MEP, a different maximal handgrip strength were observed in both groups, (NS = 20.31 vs. S = 13.96, MeanDiff = 6.35 [95%CI: 2.59–10.11], *p* = 0.001). No statistically significant differences were found in gait speed or skeletal muscle mass index (*p* = 0.156 and *p* = 0.214, respectively).

For PEF, differences were observed in gait speed (NS = 0.82 vs. S = 0.58, MeanDiff = 0.24 [95%CI: 0.32–0.45], *p* = 0.025), but not in handgrip strength (NS = 17.90 vs. S = 17.90, MeanDiff = 0.01 [95%CI: −4.99–4.98], *p* = 0.997) (Figure 3).

### 3.4. Evaluation of the Relationship between Sarcopenia Parameters and Blood Analytical Markers

No significant associations were found when analyzing the possible correlations between the parameters of the hemogram (white blood cells, hemoglobin, erythrocytes, and platelets) and the parameters of respiratory sarcopenia and somatic sarcopenia (*p* > 0.05 in all cases).

**Figure 4:**
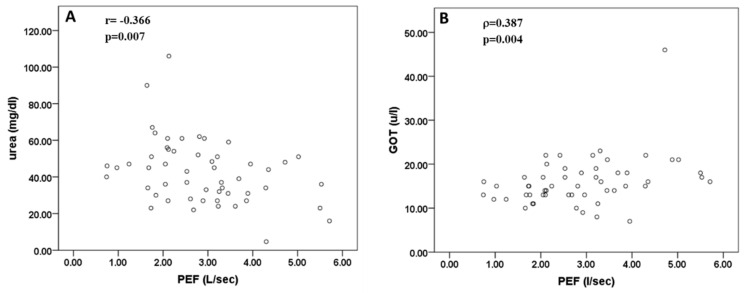
Correlation between PEF and urea (**A**) and GOT (**B**) concentration.

The relationship between respiratory sarcopenia parameters and biochemical parameters (glucose, urea, urate, cholesterol, triglycerides, creatinine, glutamic oxaloacetic transaminase [GOT], and serum glutamic pyruvic transaminase [GPT], sodium ions [Na^+^], potassium ions [K^+^], Calcium [Ca^++^]) was subsequently studied. There was a significant and positive correlation between PEF values and GOT (r = 0.387, *p* = 0.004, Spearman’s test) and a significant and negative correlation between PEF values and urea (r = −0.366, *p* = 0.007, Pearson test) (Figure 4). No significant correlation was found between other parameters of biochemical markers and respiratory sarcopenia parameters values (*p* > 0.05 in all cases, Pearson’s and Spearman’s correlation test).

We also categorized patients based on criteria of respiratory sarcopenia according to Kera et al. (22) and Ohara et al. (4) (see methods) and we evaluated whether there were any differences on blood analytical markers. 

Significant differences were found in urea values for the presence of sarcopenia estimated by PEF (NS = 32.58 vs. S = 46.70, MeanDiff = 14.12 [95%CI: −23.59–4.64], *p* = 0.005) but not in GOT values (NS = 18.50 vs. S = 14.97, MeanDiff = 3.53 [95%CI: −1.18–8.23], *p* = 0.132).

**Figure 5:**
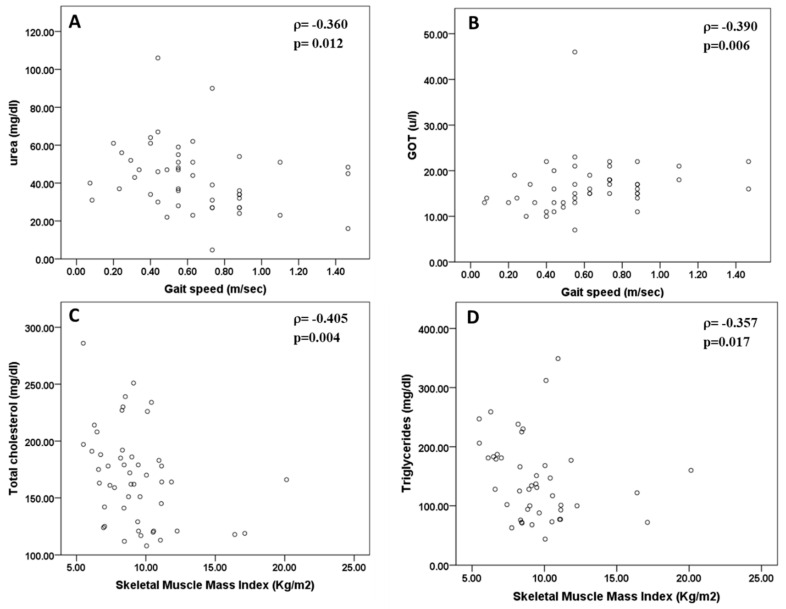
Correlation between skeletal muscle sarcopenia parameters and urea (**A**), GOT (**B**) and lipids ((**C**): total cholesterol; (**D**): triglycerides) concentration in blood.

Studying the possible correlations between somatic sarcopenia values and biochemical parameters showed a significant and positive correlation between handgrip strength and urate concentration (r = 0.279, *p* = 0.041, Spearman’s test) and between gait speed and GOT (r = 0.390, *p* = 0.006, Spearman’s test). There was also a significant and negative correlation between skeletal muscle mass index and total cholesterol (r = −0.405, *p* = 0.004, Spearman’s test) and triglycerides (r = −0.357, *p* = 0.017, Spearman’s test), and between urea and gait speed (r = −0.36, *p* = 0.012, Spearman’s test). No significant correlation was found between other parameters of biochemical markers and muscle mass and function values (*p* > 0.05 in all cases, Spearman’s correlation test) (Figure 5).

We also categorized the patients based on the cut-off scores of the three parameters studied for the evaluation of sarcopenia (see Methods section) and evaluated if there were any differences in blood analytical markers. 

For the gait speed, there were statistically significant differences in urea values (NS = 34.72 vs. S = 45.82, MeanDiff = 11.10 [95%CI: −20.44–1.76], *p* = 0.042) but not in GOT values (NS = 17.0 vs. S = 16.42, MeanDiff = 0.58 [95%CI: −2.31–3.48], *p* = 0.685).

For the presence of sarcopenia according to lean mass content, there were statistically significant differences in total cholesterol values (NS = 162.29 vs. S = 199.13, MeanDiff = 36.83 [95%CI:−71.58—2.08], *p* = 0.04) but not in tryglicerides (NS = 135.92 vs. S = 180.75, MeanDiff = 44.83 [95%CI: −99.94–10.27], *p* = 0.101)

As for handgrip strength, no differences were observed in urate values between groups (NS = 4.74 vs. S = 4.79, MeanDiff = 0.05 [95%CI: −0.94–0.85], *p* = 0.807).

## 4. Discussion

This study, which analyzes sarcopenia parameters in older people living in nursing homes, shows the direct relationship between respiratory muscle function and skeletal muscle function, especially with regard to the muscular strength and walking speed, and we report on the correlation between sarcopenia parameters and several biochemical markers obtained in routine blood analysis. This is the first study, to our knowledge, that considers the relationship between respiratory muscle strength and blood biochemical markers, finding a relationship between peak expiratory flow (PEF) values and glutamate-oxaloacetate transaminase (GOT) and urea concentration. We also observed associations between musculoskeletal parameters of sarcopenia with some blood markers, e.g., muscle mass and total cholesterol and triglyceride values, walking speed and urea and GOT values and handgrip strength and urate values. We discuss these new findings below.

The prevalence of sarcopenia in the sample of nursing home residents, following the EWGSOP criteria [2] and adjusting the skeletal muscle mass index to the Spanish population according to the cut-off points proposed by Masanés and coworkers [33], was 17.6%. These data are lower than those previously proposed for the Spanish institutionalized population [8], 41.4% applying the same assessment criteria, but are consistent with those described in a literature review that includes studies in several countries of patients residing in long-term care homes [6], like the population of our study. It is possible that the exclusion of patients who were not able to understand the content of the questionnaires influences the prevalence of the sample in the present study, since the presence of cognitive impairment increases the rates of sarcopenia [34].

The relationship between respiratory function parameters and somatic sarcopenia in community-dwelling older people has been studied in recent years, given the objectivity of these parameters and the ease and speed of assessment, but no studies in nursing home residents displaying higher levels of functional impairment and comorbidity burdens have been reported. Three parameters of respiratory function that have been established in the literature as determinants of respiratory sarcopenia, PEF [35] and maximum inspiratory (MIP) and expiratory (MEP) respiratory pressures [4].

Prevalence scores of respiratory sarcopenia according to PEF values were 70.7%, while maximum respiratory pressures were 80.7% according for MIP and 37.9% for MEP. The highest prevalence values of respiratory sarcopenia were obtained for both MIP and PEF, as in the study by Bahat et al. [36]. This may be due to the fact that loss of respiratory muscle strength occurs first in the inspiratory muscles, and is related to deterioration of type IIx and/or IIb muscle fibers of the diaphragm [3]. Loss of inspiratory muscle strength (MIP) leads to a reduced volume of inspired air prior to glottal closure and contraction of the expiratory muscle, preventing effective maximal expiration (PEF) [37,38]. In addition, it implies an inability to fully inflate the lungs, which is necessary to achieve the optimization of the length-tension relationship of the expiratory muscles, stimulate lung surfactant production and distribution, and open the collapsed peripheral airways that often accompany the hypoventilation processes associated with age and the aging process [39,40,41].

Furthermore, the greater relevance of the inspiratory muscles in the deterioration of the peripheral muscles was also justified by the decline in handgrip strength (84.5% of the sample studied) and the decline in walking speed (65.4% of the sample studied), as established in previous studies [4,19].

PEF was considered the most relevant parameter for establishing respiratory sarcopenia by Kera et al. [35,42] due to the involvement of the respiratory muscles in its execution and the minimal impact of the deterioration of the airway on its values, since it is measured at the beginning of forced expiration, and is not affected by the modifications in elastic recoil and thorax compliance associated with age [43]. The authors highlighted their preference for this test over respiratory muscle strength because of the lesser effort required and to avoid maneuvers that involve an increase in intracranial pressure, with the risks that this entails [35].

The results of this study confirm the results obtained by Kera et al. [42] in community-dwelling older people, but obtain higher values of correlation than Kera in the criterion of strength (handgrip strength) (r = 0.375 vs. r = 0.283) and in the criterion of functional performance (gait speed) (r = 0.563 vs. r = 0.167). No correlation was obtained in this study with the index of musculoskeletal mass, with muscle function more relevant than the amount of existing lean muscle mass in sarcopenic older individuals. In turn, Kera et al. [35] obtained differences between patients categorized as respiratory sarcopenic for the three determining variables of somatic sarcopenia, which were always higher in non-sarcopenic patients, while these differences were only obtained for gait speed in this study, possibly due to the high rates of sedentarism among nursing home residents and their more limited independence in their basic activities of daily life. In our study, no associations were found between respiratory muscle function and lean mass content and it could be explained in part by the obesity paradox [44]. The body mass index in the study sample widely varies among the participants enrolled in the study (range 18.7–50.2) and one third of patients have overweight and obesity grade I. This paradoxical benefit of a medically unfavorable phenotype is particularly strong in the overweight and class I obesity, and less pronounced in the more severe or morbidly obese populations (class II–III and greater). Rather than an obesity paradox, it is possible that this phenomenon may represent a “lean paradox”, in which individuals classified as normal weight or underweight may have a reduced lean mass, as a result of a progressive catabolic state and lean mass loss [45,46,47] whereas overweight and obese patients maintain an adequate lean mass content compared to under and normo-weight individuals [44,48]. Likely, the reduced respiratory muscle strength in overweight and obese individuals could be explained by other pathophysiological factors related to excessive fat accumulation in the thoracic-abdominal region which limits the chest wall expansion and diaphragm contraction, lengthens abdominal muscles, reduces the upper airway calibre, modifies airway configuration, and increases in intra-abdominal pressure and these effects may reduce respiratory muscle function independently on lean mass content [49,50,51]. Alternatively the reduced muscular function in obese individuals may be also related to chronic low-grade inflammation characterized by the predominance of interleukin-1β, interleukin-6, and tumor necrosis factor-α (TNF-α) observed in obese patients [52]. Further studies with larger sample should evaluate in details the comparison the effects of underweight, obesity with or without sarcopenia on respiratory muscle strengths in order to shed new lights on these apparent discrepancies between muscular strength and lean mass content.

Other reports suggested that valuable markers of reduced respiratory muscle strength are the values related to the maximum respiratory pressures (MIP and MEP), because these parameters are a more direct measurement of the maximum strength of respiratory muscles [4,19,36,53]. In our study, MIP correlated with both walking speed and handgrip strength (r = 0.599 and r = 0.354, respectively), while MEP correlated with handgrip strength (r = 0.465). This parameter, which is slightly more difficult to evaluate than the PEF due to its assessment procedure, is directly related to the loss of strength in the peripheral muscles, as seen in previous studies not only of older people living in the community [19,53] and in nursing homes [4], but also in healthy [54] and hospitalized young adults [55]. On the other hand, no relationship could be found between skeletal muscle mass index and maximum respiratory pressures, like those reported in previous studies of healthy older patients [53] and older patients with cardiovascular diseases [19].

These parameters of maximum respiratory strength appear to be good indicators of reduced respiratory muscle strength in older institutionalized individuals, since patients who presented sarcopenia according to these cut-off values presented significantly lower values of gait speed and handgrip strength that were as good as those recently shown by community dwelling older adults [4].

We demonstrated that parameters related to reduced respiratory muscle strength, e.g., PEF values, are significantly associated with urea and GOT concentrations in blood, which have not been previously reported for the respiratory muscle function. GOT, also known as aspartate aminotransferase, is a mitochondrial and cytoplasmic enzyme, with an important role in cell energy production [56]. Alterations in GOT levels in blood are considered well-known markers of hepatic, myocardial and skeletal muscle cytolysis, while GPT also known as alanine aminotransferase, is mainly a hepatic cytoplasmic enzyme [57,58,59]. In our study, the lack of a significant association between PEF and GPT levels in blood suggests that the association between PEF and GOT levels is related to myocardial or skeletal muscle metabolism. High serum GOT with normal serum GPT is highly prevalent among community dwelling older individuals who are underweight, and might reflect skeletal muscle pathology [60]. Furthermore, high levels of GOT in serum are present in obese subjects, regardless of age, which may be associated with sarcopenic obesity, reduced muscle mass and overweight, in some of the subjects studied [61,62]. However, the processes involved in regulating blood GOT levels in both underweight and obese subjects remain unknown, but they seem to be related to low muscle mass and function, and in this respect we found a new association with PEF values. The role of cardiac diseases cannot be ruled out, since 30% of the sample presents a comorbidity of this type. However, due to the limited size of the sample of nursing home residents with preserved cognitive function necessary to perform spirometry analysis, it was impossible to study selective pathologies.

However, confirming the association between GOT levels and muscular metabolism and function, GOT levels were also found to be significantly associated with gait speed and almost significantly with grip strength (*p* = 0.05). PEF values were also inversely and significantly associated with urea concentration in blood. Elevated serum urea, a breakdown product of protein, is generally considered a marker of muscle wasting in several conditions [63,64]. Another possible explanation for increased urea levels could be an alteration in kidney function, but the creatinine levels in our study were not significantly associated with any of the sarcopenia parameters and the correlation between urea levels and PEF therefore suggested effects based on muscle metabolism. A recent study with a machine learning approach found that urea concentration is one of risk factors for the development of predictive models for patients with sarcopenia [65], and we also reported an association between urea levels and gait speed. In relation to the positive correlation between uric acid levels and muscle strength reported in our study, this finding replicates the association reported in community dwelling-older individuals in the “InCHIANTI” study [66], which observed that higher urate levels were significantly associated with higher measures of muscle strength, and concluded that high urate levels could create a protective reaction that would counteract the excessive production of free radicals that damage muscle proteins and reduce muscle strength. Likewise, Can et al. [67], focusing on markers of inflammation and oxidative stress, analyzed a sample of 72 geriatric patients confirmed that patients with sarcopenia had significantly lower levels of uric acid than non-sarcopenic patients. Moreover, high serum urate levels are a good positive predictor of grip strength in nonagenarian older individuals, and may delay the progression of sarcopenia [68]. The skeletal muscle criterion of lean mass content was the only criterion that was significantly (and inversely) correlated with blood lipid (cholesterol and triglycerides) concentration. The aging process stimulates the appearance of fat infiltration in muscle tissue, and obesity enhances fat deposits at visceral level, in the liver, heart, pancreas and skeletal muscle, which generates a negative effect on sarcopenia. These lipids cause a pro-inflammatory effect that secretes paracrine and cytokine hormones, promoting a feedforward cycle by producing intramyocellular lipids. This toxicity generated by fats hinders the contraction of muscle fibers and the synthesis of muscle proteins, favoring the development of sarcopenia [69,70].

A study by the South Korean KNHANES conducted an evaluation of sarcopenic obesity subjects and showed a link to an increased risk of dyslipemia in these patients [71]. Mesinovic et al. [72] recently determined the associations between metabolic syndrome and components of sarcopenia, including muscle mass and quality, absolute and relative strength, and physical performance, in 84 overweight and obese older adults, and demonstrated that triglyceride levels had a negative association with leg extension strength and lower-limb relative strength. Lu et al. [73] reported that serum triglycerides and high-density lipoprotein cholesterol were independently associated with sarcopenic obesity. All the biomarkers found to be significantly associated with sarcopenia indexes can be obtained in a routine blood analysis, as they can be rapidly, inexpensively, and reproducibly assayed. Future longitudinal investigations should test these biomarkers as a part of a valuable panel of metabolites to diagnose sarcopenia and monitor the efficacy of clinical interventions in sarcopenic individuals. This is the first study to demonstrate an independent relationship between respiratory muscle strength and some aspects of body sarcopenia in institutionalized elderly people with high rates of comorbidities and polypharmacy. The fact that respiratory sarcopenia is associated with muscle strength and gait speed supports the beneficial effect of various exercises and rehabilitation interventions on breathing muscles [74,75,76]. New randomized clinical trial should evaluate the effects of such interventions not only for skeletal sarcopenia but also to improve respiratory muscle strength thus allowing a better respiratory function which can influence many respiratory tract diseases since the impairment of (inspiratory and expiratory) respiratory muscles is a common clinical finding, not only in patients with neuromuscular disease but also in those with respiratory diseases affecting the lung parenchyma or airways [77,78,79]. We provided further evidence for the use of suitable cut-off points for respiratory muscle strength which can be tested in future researches prior its proposal as indicator of muscle respiratory function in clinical settings. Loss of mass and function of the respiratory muscles could be prevented by properly applying these exercises. More studies on sarcopenia and its effects on respiratory muscle strength are needed to improve life expectancy and quality of life in the older institutionalized individuals.

## Figures and Tables

**Table 1:** Characteristics of the study sample.

Clinical and Demographic Characteristics of Participants	Mean Value ± SD (Range) or Percentage
Age (years)	78.6 ± 8.9 (55–93)
Sex	Male 32.8%Female 67.2%
IBM (kg/m^2^)	28.9 ± 6.1 (18.7–50.2)
Smokers	15.5%
Use of bronchodilators as a usual treatment	21.1%
Walking ability	Independent 63.8%Can 3.4%Walker 32.8%
Comorbidities (Charlson index)	5.4 ± 1.9 (1–11)
Diabetes	31.0%
Chronic obstructive pulmonary disease	17.2%
Hypertension	32.8%
Hypercholesterolemia	37.9%
Congestive heart failure	10.3%
Renal failure	12.1%
Osteoporosis	20.7%
Depression	19.0%

**Table 2:** Respiratory function parameters.

Respiratory Parameters	Mean Value (± SD)	Range
SatO_2_ (%)	95.9 ± 1.9	91.0–99.0
Heart rate (bpm)	77.1 ± 14.2	49.0–114.0
FVC (L/s)	1.8 ± 0.7	0.3–4.4
FEV1(L/s)	1.3 ± 0.5	0.3–2.9
FEV1/FVC (%)	76.5 ± 12.1	45.9–100.0
FEV25-75 (L/s)	1.2 ± 0.5	0.3–3.1
PEF (L/s)	2.8 ± 1.2	0.7–5.7
MIP (H_2_O cm)	36.5 ± 17.4	7.0–77.0
MEP (H_2_O cm)	58.9 ± 23.7	10.0–99.0

**Table 3:** Anthropometric analysis and sarcopenia parameters.

Anthropometric Analysis	Mean Value (± SD)	Range	% of Individuals Fulfilling the EWGSOP Criterion for Sarcopenia
Muscle mass (Janssen)	22.8 ± 8.2	13.2–49.5	Reduced lean mass: 17.6%
Skeletal muscle mass index (Janssen)	9.2 ± 2.8	5.5–20.1	Reduced lean mass: 17.6%
Hand grip in dominant hand (Kg)	17.9 ± 8.5	6.5–42.0	Muscle strength (dominant hand): 84.5%
Hand grip in non-dominant hand (Kg)	16.5 ± 7.6	3.3–36.7	Muscle strength (non-dominant hand): 84.5%
Walking speed (m/s)	0.6 ± 0.3	0.1–1.5	Physical performance: 65.4%

## Abbreviations

95% CI	95% Confidence interval
EDTA	Ethylenediaminetetraacetic acid
EWGSOP	European Working Group for Sarcopenia in Older People
FEV1	Forced Expiratory Volume in first second
FEV2575	Mesoexpiratory volume
FVC	Forced vital capacity
GOT	Glutamic oxaloacetic transaminase
GPT	Glutamic pyruvic transaminase
MEP	Maximal expiratory pressure
MIP	Maximal inspiratory pressure
NS	Non-sarcopenic individuals
PEF	Peak expiratory flow
RMS	Respiratory muscle strength
S	Sarcopenic individuals
Sat O_2_	Oxyhemoglobinic saturation
SD	Standard deviation
VC	Vital capacity
